# A Fluorescence‐Based High‐Throughput Screening Method for EGFR Inhibitors Using an EGFR‐Targeted Environment‐Sensitive Fluorescent Probe

**DOI:** 10.1155/jamc/9333993

**Published:** 2026-11-03

**Authors:** Li-Xia Wang, Xiang-Dan Cuan, Xian-Yun Chen, Xuan-Jun Wang

**Affiliations:** ^1^ School of Modern Agriculture and Food Engineering Chuzhou University Chuzhou 239000 China chzu.edu.cn; ^2^ Key Laboratory of Pu-er Tea Science Yunnan Agricultural University Ministry of Education Kunming 650201 China meb.gov.tr; ^3^ College of Agronomy Chuxiong Normal University No.546 S Rd. Lucheng Chuxiong 675099 China cxtc.edu.cn

**Keywords:** EGFR inhibitor, flavokawain A, fluorescent probe, screening

## Abstract

Fluorescence technology offers a highly sensitive, rapid, and efficient approach for drug screening at the cellular level, enabling the direct quantification of drug‐target binding interactions through precise measurement of fluorescence intensity changes. In our previous research, a small‐molecule fluorescent probe targeting epidermal growth factor receptor (EGFR) was successfully developed, featuring strong targeting specificity, low background signal, and an “off–on” response feature. Based on the EGFR‐overexpressing A431 cell line, this study established a cell‐based screening system for EGFR inhibitors and successfully identified flavokawain A (FKA), a novel potential EGFR inhibitor. FKA notably reduces the expression levels of *p*‐EGFR and its downstream molecule *p*‐Erk in the EGFR signaling pathway. It demonstrates a potent inhibitory effect on the proliferation of A431 cells, effectively suppressing clone formation and cellular migration, and exhibits a strong apoptosis‐inducing effect against A431 cells. This fluorescence‐based high‐throughput screening method has demonstrated its effectiveness in identifying novel EGFR inhibitors from a wide range of natural materials. It holds significant application potential and plays a crucial role in expediting the discovery of EGFR inhibitors from medicinal sources.

## 1. Introduction

Cancer poses a significant threat to human life and health, making its treatment and prevention a continuing challenge in the fields of life sciences and nutritional health. Molecular targeted therapy has emerged as a primary approach for cancer treatment [1, 2]. The abnormal mutation and overexpression of epidermal growth factor receptor (EGFR) significantly impact the proliferation and differentiation of various tumor cells, establishing it as an attractive target for molecular targeted therapies, especially for nonsmall cell lung cancer [3, 4]. Currently, EGFR inhibitors used in clinical practice are primarily categorized into two classes: monoclonal antibodies and small‐molecule tyrosine kinase inhibitors [5–7]. However, both types of inhibitors present specific limitations in clinical settings. For instance, monoclonal antibodies are associated with complex dosing regimens, severe side effects, and high costs, which restrict their widespread application [8]. Meanwhile, the long‐term use of small‐molecule tyrosine kinase inhibitors can lead to the development of EGFR mutations, resulting in drug resistance and ineffectiveness [9, 10]. Notably, the first‐generation EGFR tyrosine kinase inhibitor gefitinib exerts its antiproliferative activity by competitively occupying the ATP‐binding pocket of the EGFR kinase domain, thereby suppressing receptor phosphorylation and downstream signaling. Originally developed for the treatment of EGFR‐expressing lung adenocarcinoma, gefitinib initially demonstrated clinical efficacy primarily in female, nonsmoking patients with adenocarcinoma histology. However, subsequent long‐term clinical observations revealed that its in vivo selectivity is significantly diminished compared with in vitro profiles, and it frequently fails to induce responses even in tumors with high wild‐type EGFR expression. Although gefitinib shows enhanced potency against EGFR harboring the L858R activating mutation in exon 21, this genetic alteration is detected in only approximately 6% of cases. By contrast, wild‐type EGFR is expressed in roughly 60% of patients, for whom no effective targeted therapies are currently available. Therefore, the development of innovative therapeutic strategies for wild‐type EGFR‐driven malignancies remains an important unmet clinical challenge [11, 12].

Screening and exploring active molecules with inhibitory activity against EGFR from herbal medicines offers a promising approach for cancer treatment, given the abundance of various antitumor active ingredients in herbal materials, particularly those capable of significantly suppressing EGFR activation [9]. Currently, kinase activity assays, which are in vitro molecular‐level screening methods, are the primary approach for identifying EGFR inhibitors [13]. However, the limitations of in vitro screening often result in many candidate molecules demonstrating no inhibitory activity in cell lines, and the process of screening novel EGFR inhibitors from natural product libraries is constrained. Therefore, an efficient cell‐based screening system is anticipated to expedite the development of new EGFR inhibitors. Fluorescent probes, particularly small‐molecule fluorescent probes, have gained popularity in disease diagnosis and drug screening due to their high sensitivity and specificity [14, 15]. Cellular drug screening utilizing small‐molecule fluorescent probes offers a highly sensitive, rapid, and efficient method, allowing for direct evaluation of binding changes between compounds and their corresponding targets through quantitative measurement of fluorescence intensity or other parameters [16].

Over recent years, several distinct high‐throughput screening strategies have been developed for EGFR‐targeted inhibitor discovery, each offering complementary advantages and inherent limitations. Activity‐based fluorescent probes, which rely on covalent modification of the kinase active site, provide high specificity but typically require cell lysis and are incompatible with real‐time live‐cell monitoring [17]. Environment‐sensitive receptor probes, by contrast, enable direct assessment of target occupancy in living cells under physiological conditions, allowing kinetic displacement measurements without wash steps. Target‐engagement systems, such as the tetrahedral DNA nanostructure‐based FRET platform, have been successfully applied to high‐throughput screening of EGFR/Ca^2+^ signaling modulators in stem cell‐derived cardiomyocytes [18]. In parallel, unbiased genetic approaches, including genome‐wide CRISPR screening, have been employed to identify resistance mechanisms and combination therapy opportunities for EGFR inhibitors [19], while immune‐oncology high‐throughput platforms have revealed that EGFR inhibitors can enhance antigen‐specific T‐cell–mediated tumor killing through MHC‐I upregulation [20]. Among these approaches, cell‐based fluorescent probe systems offer distinct advantages in preserving native membrane architecture and receptor conformation, thereby providing physiologically relevant binding information that complements traditional in vitro kinase activity assays.

In our previous research, based on the environment‐sensitive strategy, fluorescent probe GN3 (Figure S1) was designed and synthesized by introducing fluorophores nitrobenzofurazan (NBD) to the 6‐position of gefitinib, which is capable of specifically targeting EGFR and exhibits a notable EGFR‐based “off‐on” response feature [21]. This research suggests that GN3 has potential application in the field of EGFR^wt^ inhibitor screening, motivating us to continue exploring the screening system for EGFR^wt^ inhibitors. Then, in this study, we established a cell‐based screening system using fluorescent probe GN3 for EGFR^wt^ inhibitors based on the A431 cell line, which overexpresses EGFR^wt^. The primary principle underlying this system relies on the competitive binding of EGFR inhibitors with GN3 to the ATP‐binding pocket of EGFR. When EGFR inhibitors bind to this pocket, competition would happen between EGFR inhibitors and GN3, leading to significant fluorescence quenching in cells overexpressing EGFR. Therefore, the intensity of the fluorescence signal can serve as an indicator of the competitive binding ability between a compound and GN3, which in turn reflects the compound’s binding ability to EGFR. This approach allows us to assess the potential of various compounds as EGFR inhibitors by measuring their impact on the fluorescence signal generated by GN3 in A431 cells; the schematic diagram of the screening mechanism is shown in Figure 1. By establishing this screening system, a chalcone flavokawain A (FKA), which is derived from the food‐medicine homology plant Kava, could significantly cause the fluorescence signal intensity to decrease. Then, FKA was proved to be a potential EGFR inhibitor by evaluating its inhibitory activity on the tumor cell A431, which overexpressed EGFR. These findings demonstrate that the screening platform developed in this study represents a robust tool for the rapid identification of bioactive components with EGFR inhibitory activity from herbal medicine resources. This approach establishes a novel methodology for discovering potential EGFR inhibitors from diverse natural compounds sourced from food‐medicine homologous materials, thereby offering a promising strategy to expedite the development of next‐generation EGFR‐targeting therapeutics.

**FIGURE 1:**
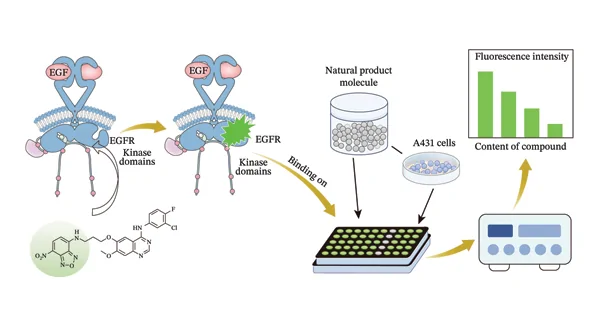
Schematic diagram of the screening mechanism.

## 2. Materials and Methods

### 2.1. Chemicals and Reagent

Fetal bovine serum (FBS) was purchased from Gibco (Invitrogen, Carlsbad, CA, USA). Penicillin–streptomycin solution (P/S), EDTA, Dulbecco’s modified Eagle’s medium phenol red‐free medium, and RPMI‐1640 medium were obtained from Meilunbio (Dalian, China). Molecular biology‐grade dimethyl sulfoxide (DMSO) was purchased from VWR life science (Atianta, GA, USA). CellTiter 96 AQueous One Solution Cell Proliferation Assay (MTT) was purchased from Solarbio (Beijing, China). Antibodies against EGFR (D38B1) (cat. no. 4267), *p*‐EGFR (Tyr‐1068) (cat. no. 2234), P44/42MAPK/Erk (cat. no. 9194), and p‐P44/42MAPK/Erk (cat. no. 9121) were obtained from Cell Signaling Technology (Beverly, MA, United States). The antibody against GAPDH (cat. no. 10094‐MM09) was purchased from Sino Biological Inc. (Beijing, China). Drugs of gefitinib, lapatinib, paclitaxel, etoposide, capecitabine, and 5‐fluorouracil were purchased from Shanghai Yuanye Bio‐Technology Co., Ltd. (Shanghai, China). The natural compounds were purchased from BioBioPha Co., Ltd. (Kunming, China).

### 2.2. Cell Culture

A431 cells were obtained from the Kunming Cell Bank of the Chinese Academy of Sciences (Kunming, China) and cultured in complete DMEM containing 10% FBS and 1% penicillin/streptomycin.

### 2.3. Determination of Probe Concentration and Incubation Time

A431 cells were seeded at 1.5 × 10^4^ cells per well in black transparent‐bottom 96‐well plates and cultured overnight. After removing the supernatant and washing twice with PBS, the cells were treated with different concentrations of probe GN3 (0, 1, 2, 4, 6, 8, and 10 µM), which dissolved in 200 µL phenol‐red‐free medium for 0, 5, 10 min, until 90 min, then the fluorescence intensities were recorded at λex = 469 nm and λem = 516 nm on a microplate reader (FlexStation 3; Molecular Devices, Sunnyvale, CA, United States).

### 2.4. Effect of Exogenous EGF on Fluorescence Response

A431 cells were seeded at 1.5 × 10^4^ cells per well in black transparent‐bottom 96‐well plates and cultured overnight, then stimulated with 10 ng/mL EGF for 5, 10, 15, 20, or 25 min. After removing the supernatant and washing twice with PBS, 200 µL of phenol‐red‐free medium containing 6 µM of GN3 was added to each well for 60 min, then the fluorescence intensities were recorded at λex = 469 nm and λem = 516 nm on a microplate reader.

### 2.5. Cell Viability Assay

Cells were seeded at 1.5 × 10^4^ cells per well in 96‐well plates and cultured overnight, then treated with different concentrations of compounds for 24 h. Next, 20 μL of MTT (5 mg/mL) was added to each well, and the cells were incubated for another 4 h at 37°C. After removing the supernatant, 150 μL of DMSO was added to the wells, and the absorbance at 492 nm was recorded on a microplate reader (FlexStation 3; Molecular Devices, Sunnyvale, CA, United States).

### 2.6. Western Blotting Assay

For western blotting analysis, cells ± compound treatment were lysed in lysis buffer for 30 min on ice, and the lysates were collected. Protein samples containing equal amounts of proteins, determined with an Enhanced BCA Protein Assay Kit, were resolved by SDS–PAGE using an 8% gel before transferring to polyvinylidene fluoride (PVDF) membranes. Then, the membranes were blocked with 5% BSA at room temperature for 60 min, probed with EGFR primary antibodies overnight at 4°C, and incubated with HRP‐conjugated secondary antibodies for 60 min at room temperature. HRP was detected using a Pro‐light HRP Chemiluminescent Kit (Tiangen Biotech, Beijing, China) and a chemiluminescence detection system (FluorChem E System; ProteinSimple, Santa Clara, CA, United States).

### 2.7. Colony Formation and Migration Assay

Cells were seeded at 3000 cells per dish (60 mm) and cultured overnight. Then the cells were cultured and treated with different concentrations of compounds (0, 0.25, 0.5, 1, and 2 µM) in RPMI 1640, including 3% FBS and 1% P/S. The medium containing compounds was changed every three days, and the formation of the clones was observed under a microscope every day. When the cells grew to visible clones (the twelfth day), the culture medium was discarded and washed twice with PBS, the cells were fixed with 4% paraformaldehyde at room temperature for 20 min, the paraformaldehyde was discarded and washed with PBS, and then 0.1% crystal violet staining solution for 20 min, and the staining solution was discarded. Wash away the staining solution without staining cells in slowly running water. The cells were then dried naturally and photographed, and the results were recorded. After photography, 1 mL of 10% acetic acid was added to each dish, the stained crystal violet was completely dissolved, and then the solution was transferred to an enzyme plate. The absorption value was measured at 560 nm, and the value of the absorption was used to indirectly quantify the strength of clone formation ability.

### 2.8. Cell Apoptosis Experiment

Apoptosis of A431 cells was analyzed by flow cytometry. Cells were seeded in 6‐well plates with different concentrations of FKA (0, 2.5, 5, 10, and 20 µM) for 15h. After, cells were obtained and washed twice with precooled PBS and resuspended in 100 µL of 1 × binding buffer, and then 5 μL Annexin VFITC and 10 μL of 7‐AAD (Meilunbio, Dalian, China) were added mixed gently and incubated for 15 min at room temperature (25°C) in the dark. After staining incubation, 400 μL of 1 × binding buffer working solution was added to each tube, mixed, and detected using a BD FACSCaliburTM flow cytometer within 1 h.

### 2.9. Surface Plasmon Resonance (SPR) Analysis

SPR studies were carried out using a Biacore S200 instrument. EGFR protein (aa 668–1210) was immobilized on the Series S CM5 Sensor Chip using the standard amine‐coupling method. Immobilization was performed using an amine‐coupling kit (GE Healthcare) following the manufacture’s protocol. The analyte of FKA (0, 3.125, 6.25, 12.5, 25, 50, and 100 μM) was injected and passed over the chip surface. The kinetics and affinity assay were determined at a flow rate of 30 μL/min using PBS‐P buffer (20 mM phosphate buffer, 2.7 mM KCl, 137 mM NaCl, and 0.05% [v/v] P20 surfactant). The Kd values were calculated with the kinetics and affinity analysis option of Biacore S200 Evaluation Software Version 1.1 (GE Healthcare) [21].

### 2.10. Molecular Docking Study

The crystal structure of EGFR (PDB ID: 6jxt) [22] was obtained from the Protein Data Bank (https://www.rcsb.org/). AutoDockTools was used for the modeling of the ligand FKA and receptor EGFR. AutoDock Vina was used for grid and docking. Docking parameters were set as the default values, except “The Number of GA Runs,” which was set to 100, and the maximum number of medium was set to 5,000,000 in AutoGrid 4.2 and Autodock 4.2. The highest populated cluster with the lowest energy conformation based on the scoring function was selected as the binding mode. The conformation with the lowest energy was selected for visual analysis using PyMol v2.3.0.

### 2.11. Statistical Analysis

Graphs are presented as mean ± SEM, and experimental data were plotted using GraphPad Prism 8 (GraphPad Software, CA, USA). Statistical analysis was performed using SPSS 23.0 (one‐way ANOVA, IBM, YK, USA). *p* value < 0.05 was considered statistically significant (^∗∗∗^ *p* < 0.001, ^∗∗^ *p* < 0.01, ^∗^ *p* < 0.05). Each experiment was repeated three times.

## 3. Results and Discussion

Currently, the screening of EGFR inhibitors primarily involves the use of small molecules through in vitro kinase assays, which exhibited certain limitations as it employs kinase domain fragments rather than full‐length proteins. Furthermore, this screening is performed outside of a live cellular environment, which often leads to many candidate molecules being ineffective in subsequent experiments conducted in live cells. Fluorescent probes, besides their target detection and imaging capabilities, have garnered considerable attention in the field of drug screening [23]. Due to their high sensitivity and ease of operation, they allow for direct assessment of binding changes between drugs and their corresponding targets by quantitatively reading parameters such as fluorescence intensity. Currently, numerous research achievements have been made in the screening of enzyme activators and inhibitors using this method [24–26]. Because receptor targets lack specific reaction sites, it is necessary to rationally design probe molecules with high sensitivity, strong target specificity, and a certain “off‐on” response mechanism to establish a cell‐level screening system for receptor modulators [27, 28].

Our previous research work has successfully developed a small‐molecule fluorescent probe GN3, which specifically targets EGFR and exhibits a significant “off‐on” fluorescence response feature based on EGFR binding. This innovative probe demonstrates promising applications in the screening of EGFR inhibitors [21]. Therefore, this study successfully established a cell‐level EGFR inhibitor screening system based on the competitive binding mechanism of fluorescent probes, which was effectively used for the screening of natural small‐molecule active compounds. The fundamental principle of this system lies in the competitive binding interactions between EGFR inhibitors and the ATP‐binding pocket of EGFR, which subsequently reduces the binding affinity of the probe. When the probe dissociates from its binding site and exits the hydrophobic cavity, it results in substantial fluorescence quenching, leading to a decrease in fluorescence signal intensity. Thus, the intensity of the fluorescence signal can serve as an indicator of the competitive binding ability of various compounds to the probe, indirectly reflecting their binding affinity to the ATP‐binding domain of EGFR. This system operates within the 96‐well plate format, offering sufficient convenience and efficiency. Using the established screening system, a chalcone compound known as FKA, derived from the medicinal and edible plant *Piper methysticum* (commonly referred to as kava pepper), was identified as a potential EGFR inhibitor. These results confirmed that constructing EGFR inhibitors at the cellular level using fluorescent probes is feasible.

### 3.1. Establishment of the Fluorescent Probe‐Based Screening System for EGFR Inhibitors

#### 3.1.1. Establishment of a Screening System Based on Competitive Binding Principle

The screening principle for receptor modulators utilizing fluorescent probes is based on the competitive displacement of a probe from the receptor’s ligand binding site by a modulator [16, 29, 30]. This displacement leads to changes in the fluorescence signal, which serves as an indirect measure of the affinity between the modulator and the receptor [31, 32]. Most EGFR inhibitors are classified as ATP‐competitive binding inhibitors, bind competitively to the ATP‐binding pocket within the kinase domain, effectively blocking the transfer of the γ‐phosphate group from ATP to tyrosine residues [33]. The rationale for establishing this screening system hinges on the competition between EGFR inhibitors and the fluorescent probe GN3 for the ATP hydrophobic pocket. When EGFR inhibitors bind to this site, GN3 is unable to effectively attach to EGFR and remains unbound within the cell.

#### 3.1.2. Determination of Relevant Parameters in the Screening System

When A431 cells were treated with varying concentrations of GN3 (0, 1, 2, 4, 6, 8, and 10 µM), the fluorescent response curve was obtained, which demonstrated that the intensity plateaued at a GN3 concentration of 6 µM (Figure 2A). To establish the appropriate incubation time for the probe, we measured the fluorescence intensity at different treatment durations, using a cell count of 3 × 10^4^ per well. The results showed that as the incubation time increased, the fluorescence intensity stabilized around 60 min (Figure 2B). Therefore, 6 µM was determined to be the optimal probe concentration for the screening system, and 60 min was chosen as the optimal incubation time for the probe.

**FIGURE 2:**
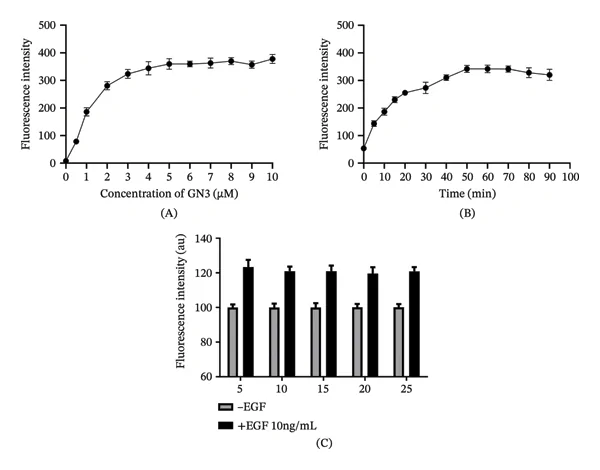
Fluorescence intensity under different conditions (λex = 469 nm and λem = 516 nm). (A) Fluorescence intensity of probe GN3 with different concentrations; (B) fluorescence intensity of probe GN3 (6 µM) at different times; (C) fluorescence intensity of probe GN3 with or without EGF (10 ng/mL); the fluorescence intensity was normalized, and the relative fluorescence intensity of the EGF‐untreated group was 100.

EGF is the natural ligand of EGFR, and the addition of exogenous EGF would stimulate excessive activation of EGFR [34]. GN3, as an EGFR inhibitor with fluorescent properties, functions similarly to gefitinib. Therefore, excessively activated EGFR can bind to more small molecules, leading to the speculation that EGF would induce the enhancement of fluorescence intensity of GN3. In this experiment, the fluorescence signals of GN3 were tested in the presence or absence of EGF at a concentration of 10 ng/mL. As shown in Figure 2C, the result indicated that exogenous EGF stimulation of excessive EGFR activation could enhance the fluorescence signal response of GN3, thereby improving the sensitivity of the screening system. The EGF‐induced stimulation times were 5, 10, 15, 20, and 25 min, respectively, and different treatment times showed little impact on the signal intensity. In the subsequent screening experiments, we chose a stimulation time of 10 min with EGF for subsequent experiments.

#### 3.1.3. EGFR Inhibitors Gefitinib and Lapatinib, Together With Five Chemotherapy Drugs, Were Used to Validate the Screening System

Gefitinib and lapatinib are classic examples of first‐generation reversible EGFR inhibitors. We conducted competitive binding experiments with GN3 using various concentrations of gefitinib and lapatinib, while simultaneously stimulating EGFR activation with EGF (10 ng/mL). The results showed that as the concentrations of gefitinib and lapatinib increased, the fluorescence signal intensity decreased significantly, exhibiting a downward trend that correlated with the inhibitor concentrations (Figure 3A,B). This indicates that the EGFR inhibitors gefitinib and lapatinib can effectively block the binding of GN3 to the hydrophobic pocket of EGFR, resulting in a reduction in fluorescence signal intensity, which can be quantitatively assessed. These findings confirm the feasibility of the screening system for EGFR inhibitors established in this study.

**FIGURE 3:**
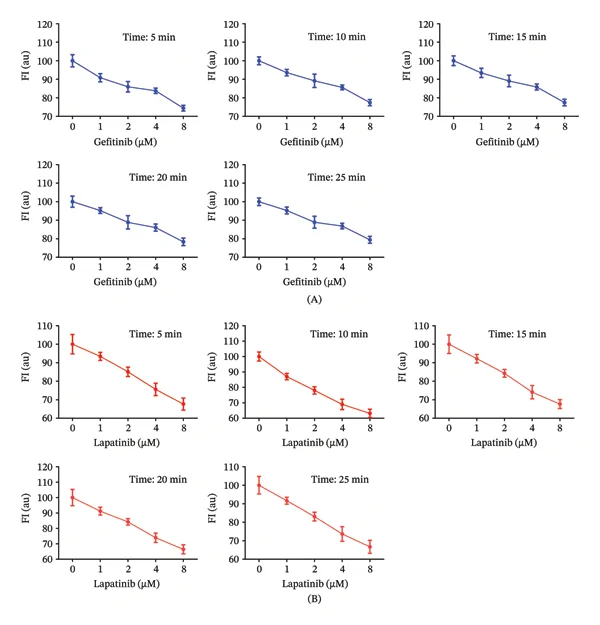
Fluorescence response curve of gefitinib (A) and lapatinib (B) competitive with GN3. Note: concentration of GN3: 6 µM; concentration of drugs: 0, 1, 2, 4, 8 µM; treated time: 60 min; the fluorescence intensity was normalized, and the relative fluorescence intensity of the control group (concentration of probe and drug was 6 and 0 µM, respectively) was 100.

To further verify that EGFR inhibitors can reduce the fluorescence intensity of GN3, while non‐EGFR inhibitors do not affect fluorescence intensity, we selected five antitumor drugs—paclitaxel, camptothecin, etoposide, 5‐fluorouracil, and capecitabine [35]—for a selective characterization of their competitive binding with GN3. As shown in Figure 4, none of the five drugs caused a decrease in fluorescence signal intensity, confirming that non‐EGFR inhibitors do not interfere with the binding of probe molecules to EGFR and significantly alter fluorescence intensity. This experiment further demonstrated that molecules that specifically bind to EGFR proteins can competitively bind to EGFR with the probe, leading to a decrease in probe signal intensity, while non‐EGFR inhibitors do not cause changes in fluorescence intensity, indicating that the probe GN3 showed high sensitivity and could be used to establish a screening system for EGFR inhibitors at the cellular level.

**FIGURE 4:**
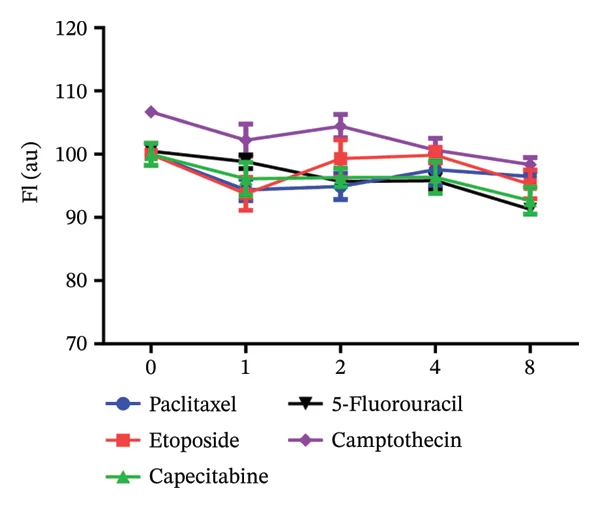
Fluorescence response curve of five chemotherapy drugs competitive with GN3. Note: concentration of GN3: 6 µM; concentration of drugs: 0, 1, 2, 4, 8 µM; treated time: 60 min; the fluorescence intensity was normalized, and the relative fluorescence intensity of the control group (concentration of probe and drug was 6 and 0 µM, respectively) was 100.

#### 3.1.4. Establishment of the Cell‐Based Screening System for EGFR Inhibitors Employing the Fluorescent Probe GN3

The aforementioned experiments demonstrated that EGFR inhibitors can competitively bind to EGFR with GN3, resulting in a decrease in fluorescence intensity. Building upon this competitive binding mechanism, a GN3‐based screening system for EGFR inhibitors was established. This screening system utilizes A431 cells as the model cell line, which display significant overexpression of EGFR, and employs black, transparent‐bottom 96‐well plates for cell culture. The specific protocol involves preseeding A431 cells at a density of 30,000 cells per well into the black, transparent‐bottom 96‐well plates and incubating overnight. With the probe concentration fixed at 6 µM, the probe is co‐incubated with varying concentrations of test compounds (0, 1, 2, 4, and 8 µM) for 60 min. Control wells without cells are also included. Following drug incubation, the cells are stimulated with exogenous EGF at a concentration of 10 ng/mL to rapidly phosphorylate EGFR, followed by an additional 10‐min incubation period. Subsequently, fluorescence intensity is measured directly (λex = 469 nm; λem = 516 nm). Data analysis was conducted using GraphPad Prism 5 to generate competitive binding curves between the probe GN3 and the tested compounds, quantitatively reflecting the compounds’ competitive binding affinities relative to GN3.

### 3.2. Screening Natural Compounds Using the Established Screening System

Our laboratory has established a natural product library comprising 1200 small‐molecule compounds aimed at the development of natural EGFR inhibitors. Initially, we screened 40 phenolic and terpenoid small molecules from this library using the established screening system; the list of natural products is presented in Table S1. As illustrated in Figure S2, one particular chalcone compound, FKA (compd. 39), markedly decreased fluorescence signal intensity, indicating that FKA may represent a potential EGFR inhibitor.

### 3.3. FKA Significantly Inhibits the Proliferation of A431 Cells

To further validate whether FKA exerts antiproliferative biological effects on A431 cells through the inhibition of the EGFR signaling pathway, we initially conducted a MTT assay to evaluate the antiproliferative activity of FKA against A431 cells. Preliminary results, as illustrated in Figure 5A, indicated that FKA significantly inhibits the proliferation of A431 cells in a concentration‐ and time‐dependent manner. Specifically, the IC_50_ values after 24 and 48 h of treatment were determined to be 6.86 ± 1.38 μM and 4.68 ± 1.43 μM, respectively. The colony formation ability of tumor cells in vitro serves as an important indicator of their tumorigenicity. To further validate the antitumor effects of FKA, this experiment assessed its impact on the colony formation capability of A431 cells. Specifically, 10,000 cells were plated in dishes and continuously treated with FKA at concentrations of 0, 2.5, 5, 10, and 20 μM for a duration of 10 days. Afterward, crystal violet staining was performed, and photographs were taken to facilitate a quantitative analysis of the number of cell colonies formed. As demonstrated in Figure 5B,C, treatment of A431 cells with varying concentrations of FKA significantly inhibited their clonogenic ability compared to the control group, with the inhibitory effect becoming more pronounced as the drug concentration increased (*p* < 0.001). These findings further confirm that FKA effectively inhibits the proliferation of A431 cells.

**FIGURE 5:**
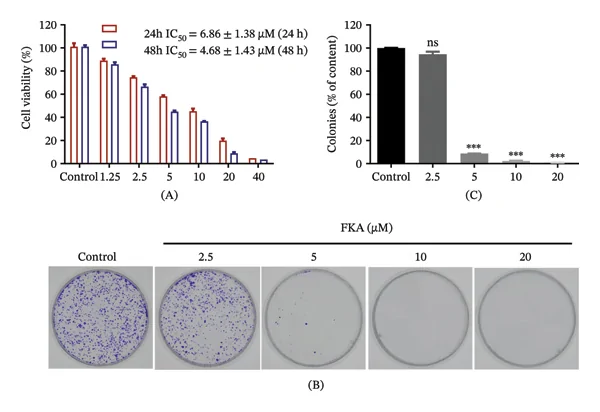
The inhibitory effect of FKA treatment in A431 cells. Note: (A) the effect of different concentrations of FKA (0, 1.25, 2.5, 5, 10, 20, and 40 μM) and EGF (10 ng/mL) on cell viability. (B) A431 cells were treated with FKA (concentrations: 0, 2.5, 5, 10, and 20 μM). (C) Quantitative analysis of clone formation. The data in the quantitative analysis chart are the average of 3 independent experiments (mean ± SD, ns = no significant, ^∗^ *p* < 0.05, ^∗∗^ *p* < 0.01, and ^∗∗∗^ *p* < 0.001).

### 3.4. FKA Significantly Inhibits the Migration of A431 Cells

A wound healing assay was conducted to evaluate the effect of FKA on the migratory ability of A431 cells. The A431 cell line was treated with FKA at concentrations of 0, 2.5, 5, 10, and 20 μM for durations of 24 and 48 h. The widths of the cellular wounds in each treatment group were observed under a microscope, and photographs were captured for documentation. The experimental results are presented in Figure 6. After 24 h of FKA treatment, minimal migration was observed in the drug‐treated groups, while the healing rate in the control group significantly increased. At the 48‐h mark, cells treated with higher concentrations of FKA exhibited almost complete inhibition of migration, whereas the control group demonstrated nearly complete wound healing. These findings indicate that FKA significantly impedes the migration of A431 cells across both treatment durations.

**FIGURE 6:**
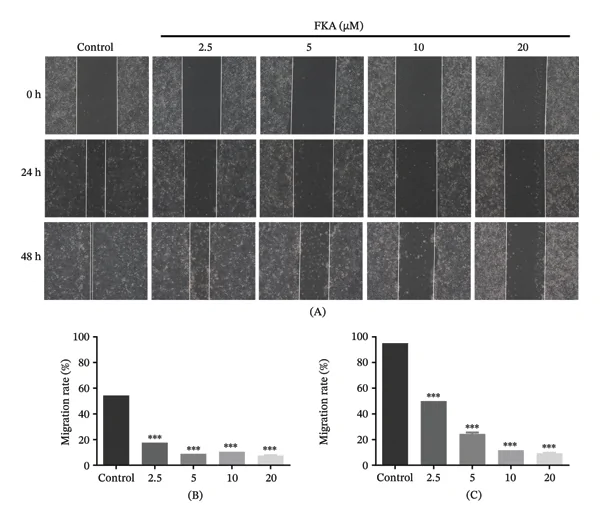
Migration of A431 cells treated with FKA. (A) The effect of migration of A431 cells treated by FKA (concentrations: 0, 2.5, 5, 10, and 20 μM); (B) quantitative analysis of migration rate treated for 24 h; (C) quantitative analysis of migration rate treated for 48 h; the data in the quantitative analysis chart are the average of 3 independent experiments (mean ± SD, ns = no significant, ^∗^ *p* < 0.05, ^∗∗^ *p* < 0.01, and ^∗∗∗^ *p* < 0.001).

### 3.5. FKA Induces the Apoptosis of A431 Cells

Apoptosis, a form of programmed cell death, is a highly regulated and energy‐dependent process that plays crucial roles in tissue homeostasis, embryonic development, and immune system regulation [36]. To further evaluate the inhibitory effects of FKA on A431 cells, this study investigated the impact of FKA on apoptosis in A431 cells using flow cytometry. A431 cells were treated with different concentrations of FKA (0, 2.5, 5, 10, and 20 µM) for 24 h, followed by staining with an Annexin V‐FITC/PI apoptosis detection kit. The apoptotic status of the various treatment groups was assessed using a flow cytometer. As shown in Figure 7, the apoptosis rate of A431 cells treated with FKA was significantly increased compared to the control group, and this increase in apoptosis was positively correlated with the concentration of FKA (*p* < 0.001). These results indicate that FKA can induce apoptosis in A431 cells in a dose‐dependent manner.

**FIGURE 7:**
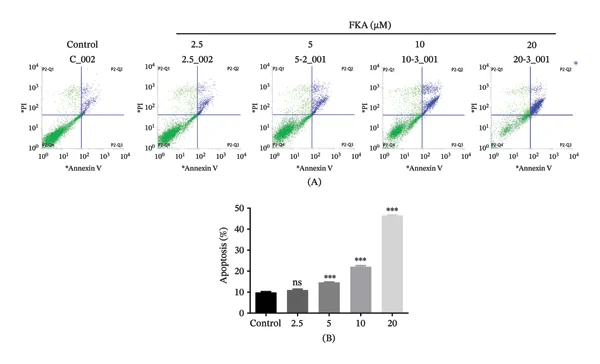
Effect of FKA on apoptosis in A431 cells. Note: (A) cell flow cytometer detects the effect of FKA (concentration: 0, 2.5, 5, 10, and 20 μM) on A431 cells after 24 h of treatment. (B) Quantitative analysis chart of cell apoptosis rate, with the control group (without drug treatment) as the control. The data in the quantitative analysis chart are the average of 3 independent experiments (mean ± SD, ns = no significant, ^∗^ *p* < 0.05, ^∗∗^ *p* < 0.01, and ^∗∗∗^ *p* < 0.001).

### 3.6. FKA Inhibits the EGFR Signaling Pathway

As a classic EGFR‐TKI, the main mechanism of action involves inhibiting EGFR phosphorylation. This experiment conducted a detailed investigation into the effects of FKA on the phosphorylated EGFR (*p*‐EGFR) expression levels and the phosphorylation levels of the Erk protein in the EGFR downstream signaling pathway under various treatment durations (1 and 4 h) and concentrations (0, 2.5, 5, 10, 20 μM). The experimental results are presented in Figure 8. It can be observed that, under stimulation with 10 ng/mL of EGF and after drug treatment durations of 1 and 4 h, FKA significantly inhibited the expression levels of p‐EGFR and p‐Erk in A431 cells, exhibiting a dose‐dependent relationship. Simultaneously, FKA did not affect the total protein levels of EGFR and Erk. These results indicate that FKA can inhibit the proliferation of A431 cells by suppressing the activation of the EGFR signaling pathway.

**FIGURE 8:**
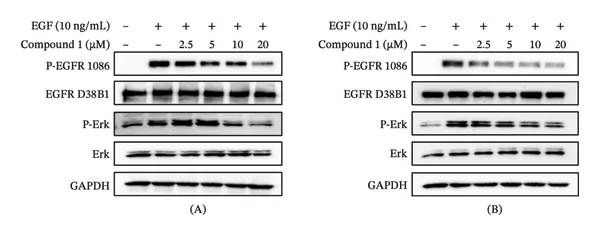
FKA reduced EGFR expression in A431 cells. Note: (A) EGF treated for 10 min, drug treated for 1 h; (B) EGF treated for 10 min, drug treated for 4 h; drug concentration: 0, 2.5, 5, 10, 20 μM; −: untreatment; +: drug treatment.

### 3.7. SPR Studies Between FKA and EGFR

In order to confirm whether FKA directly interacts with EGFR, we evaluated their real‐time interaction using a Biacore S200 SPR system. FKA exhibited binding to EGFR with fast kinetics of association and dissociation, as shown in Figure 9. For FKA at concentrations of 3.1, 6.3, 12.5, 25, 50, and 100 μM, the response reached 3.0, 5.5, 11.9, 25.5, 54.8, and 119.4 RU, respectively. The software fitting equilibrium dissociation constant (Kd) value of FKA was 4.249 × 10^−6^ M. Although the RU value of FKA was lower than those of gefitinib‐EGFR (Kd = 2.518 × 10^−6^ M) [21], it was still proved that FKA exhibited a significantly strong affinity with EGFR and could directly bind to EGFR.

**FIGURE 9:**
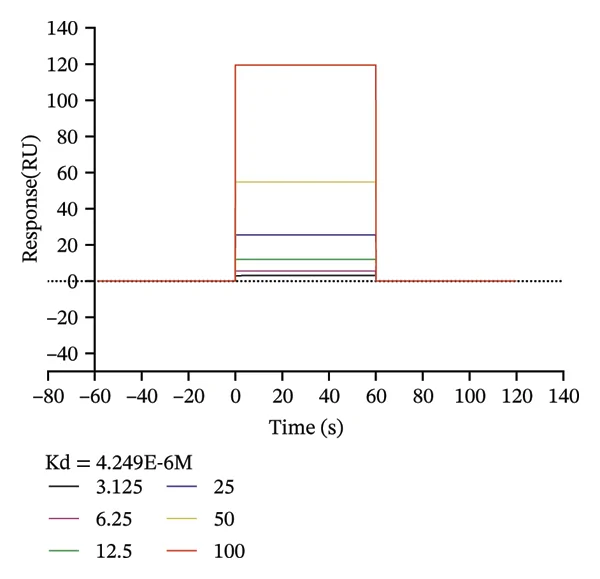
SPR sensograms for FKA binding to EGFR, at concentration of 3.125 μM (black), 6.25 μM (purple), 12.5 μM (green), 25 μM (blue), 50 μM (yellow), and 100 μM (red).

### 3.8. Molecular Docking Analysis

Molecular docking was performed to explore the potential binding mode and interaction reliability between FKA and EGFR. As illustrated in Figure 10, FKA occupies the binding pocket of EGFR with high structural complementarity. The calculated binding energy of −7.0 kcal/mol (Table S2) suggests a strong binding affinity under physiological conditions. According to established criteria [37], docking energy values below −4.25, −5.0, and −7.0 kcal/mol represent moderate, good, and strong binding affinities, respectively. Further analysis revealed that FKA forms a hydrogen bond with the key residue LYS‐745 (lysine) in the EGFR binding site, which is the main binding site of EGFR TKIs such as gefitinib. This interaction not only enhances the binding affinity between EGFR and FKA but also stabilizes the protein–ligand complex, potentially playing a critical role in modulating EGFR’s functional activity.

**FIGURE 10:**
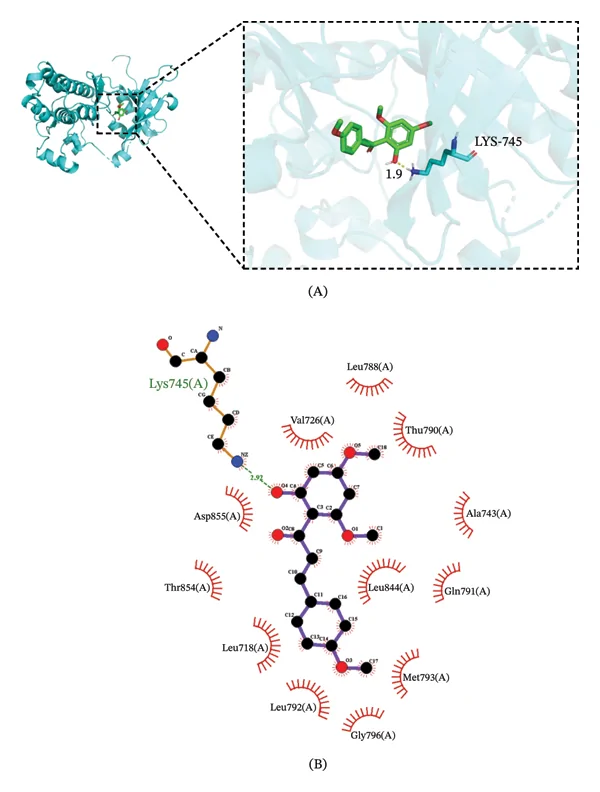
Molecular modeling of EGFR–FKA. (A) Docked structure of EGFR–FKA; (B) 2D model of FKA interaction with EGFR.

## 4. Conclusion

In this study, a screening system for EGFR inhibitors based on the fluorescent probe GN3 was established in A431 cells overexpressing EGFR. Forty naturally derived phenolic and terpene compounds were screened, and it was found that a chalcone compound, FKA, significantly caused a decrease in the fluorescent signal of the probe. Further bioactivity studies have revealed that FKA is a potential EGFR inhibitor. The novel screening method for EGFR inhibitors developed in this study can be widely applied to the screening of natural small‐molecule compounds and plant extracts. This study has successfully established a novel high‐throughput screening platform for EGFR inhibitors utilizing fluorescent probe technology, demonstrating substantial potential for broad biomedical applications. Through the strategic implementation of these probes with unique photophysical properties, the system markedly improves both the sensitivity and specificity in detecting potential EGFR inhibitors across diverse cellular contexts. These methodological innovations not only provide deeper insights into the molecular mechanisms underlying EGFR‐mediated signaling cascades but also facilitate the identification and development of targeted therapeutic agents for EGFR‐associated malignancies. Moreover, the inherent adaptability of this screening paradigm enables its potential extension to other molecular targets, thereby significantly enhancing its value in pharmaceutical research and therapeutic development across various pathological conditions.

## Funding

This study was supported by the Yunling Scholarship XDYC‐YLXZ‐2022‐0025; Scientific Research Starting Foundation of Chuzhou University, 2024qd37.

## Consent

Informed consent was obtained from all participants involved in the study.

## Conflicts of Interest

The authors declare no conflicts of interest.

## Supporting Information

Additional supporting information can be found online in the Supporting Information section.

## Supporting information


**Supporting Information** Supporting information for this article is available in SI. Figure S1: Structure of probe GN3. Figure S2: Fluorescence response curves of compounds competitive with GN3. Table S1: The structures and sources of 40 natural small molecule compounds. Table S2: Docking results of EGFR‐FKA interactions.

## Data Availability

The data that support the findings of this study are available on request from the corresponding authors. The data are not publicly available due to privacy or ethical restrictions.
