# Supplementary material for: A Fluorescence‐Based High‐Throughput Screening Method for EGFR Inhibitors Using an EGFR‐Targeted Environment‐Sensitive Fluorescent Probe
Source: J Anal Methods Chem. 2026 Nov 3;2026:9333993. doi: 10.1155/jamc/9333993 (PMC13732097; doi:10.1155/jamc/9333993)
Supplement: Supplementary file 1 [file JAMC-2026-9333993-s001.zip › Supplementary Figure and Table Legends.docx]

**Supplementary Figure and Table Legends**

Figure S1. Structure of probe GN3

Figure S2. Fluorescence response curves of compounds competitive with GN3

Table S1 The structures and sources of 40 natural small molecule compounds

Table S2 Docking results of EGFR-FKA interactions
