# Supplementary material for: A Fluorescence‐Based High‐Throughput Screening Method for EGFR Inhibitors Using an EGFR‐Targeted Environment‐Sensitive Fluorescent Probe
Source: J Anal Methods Chem. 2026 Nov 3;2026:9333993. doi: 10.1155/jamc/9333993 (PMC13732097; doi:10.1155/jamc/9333993)
Supplement: Supplementary file 1 [file JAMC-2026-9333993-s001.zip › JAMC-Supporting Information-wlx-0916.docx]

Supporting Information to Accompany “**A Fluorescence-Based High-Throughput Screening Method for EGFR inhibitors Using an EGFR-Targeted Environment-Sensitive fluorescent probe**”

Li-Xia Wang a,c*, Xiang-Dan Cuan c, Xian-yun Chen b, Xuan-Jun Wang b*
a School of Biological and Food Engineering, Chuzhou University, Chuzhou, 239000, China.
b College of agronomy, Chuxiong Normal University; No.546 S Rd. Lucheng, Chuxiong, 675099, China.
c Key Laboratory of Pu-er Tea Science, Ministry of Education, Yunnan Agricultural University, Kunming, 650201, China.
*Corresponding author: Li-Xia Wang (wanglixia0315@163.com), Xuan-Jun Wang (xjwang@cxtc.edu.cn)

Figure S1. Structure of probe GN3

Table S1 The structures and sources of 40 natural small molecule compounds

| Compound No. | Name | Structure | Main sources |
| --- | --- | --- | --- |
| Compd.1 | 3-Epikatonic acid |  | *Tripterygium wilfordii* |
| Compd.2 | Olean-12-ene-3,24-diol |  | *Salvia japonica* |
| Compd.3 | Bryonolic acid |  | *Siraitia grosvenorii*  *Coriaria nepalensis* |
| Compd.4 | 22-Hydroxy-3-oxoolean-12-en-29-oic acid |  | *Punica granatum* |
| Compd.5 | Scillascillol |  | *Scilla scilloides* |
| Compd.6 | Scillascillone |  | *Scilla scilloides* |
| Compd.7 | 15-Deoxoeucosterol |  | *Scilla scilloides* |
| Compd.8 | Scillascilloside B-1 |  | *Scilla scilloides* |
| Compd.9 | Cycloart-25-ene-3β-24-diol |  | *Tillandsia usneoides* |
| Compd.10 | Spinasterol glucoside |  | *Arenaria brevipetala* |
| Compd.11 | Ergosterol |  | various edible fungi |
| Compd.12 | 1-O-Deacetylkhayanolide E |  | *Swietenia mahagoni* |
| Compd.13 | Arteannuin B |  | *Artemisia annua* |
| Compd.14 | Deoxyartemisinin |  | *Artemisia annua* |
| Compd.15 | Oplopanone |  | *Hedychium yunnanense*  *Schisandra chinensis* |
| Compd.16 | Mucrolidin |  | *Homalomena occulta* |
| Compd.17 | Homalomenol A |  | *Homalomena occulta* |
| Compd.18 | Oplodiol |  | *Homalomena occulta* |
| Compd.19 | 6,8-Cyclo-1,4-eudesmanediol |  | *Magnolia liliflora* |
| Compd.20 | 1,4,7-Eudesmanetriol |  | *Magnolia liliflora* |
| Compd.21 | Coriatin |  | *Coriaria nepalensis* |
| Compd.22 | 6-O-Acetylcoriatin |  | *Coriaria nepalensis* |
| Compd.23 | Tutin |  | *Coriaria nepalensis* |
| Compd.24 | Plumieride |  | *Plumeria rubra* |
| Compd.25 | Norlichexanthone |  | *Scilla scilloides* |
| Compd.26 | Silybin |  | *Silybum marianum* |
| Compd.27 | Naringin |  | *Citrus reticulata* |
| Compd.28 | Tephrosin |  | *Derris trifoliata* |
| Compd.29 | Sophpricoside |  | *Sophora japonica* |
| Compd.30 | Drimiopsin D |  | *Scilla scilloides* |
| Compd.31 | Drimiopsin C |  | *Scilla scilloides* |
| Compd.32 | 2-Hydroxy-7-O-methylscilla |  | *Scilla scilloides* |
| Compd.33 | Scillascillin |  | *Scilla scilloides* |
| Compd.34 | 4′-Demethyl-3,9-dihydroeucomin |  | *Scilla scilloides* |
| Compd.35 | 3′-Hydroxy-3,9-dihydroeucomin |  | *Scilla scilloides* |
| Compd.36 | Arbutin |  | *Bergenia purpurascens* |
| Compd.37 | Resveratrol |  | *Vitis vinifera* |
| Compd.38 | Gastrodin |  | *Gastrodia elata* |
| Compd.39 | Flavokawain A |  | *Piper methysticum* |
| Compd.40 | Pinoresinol |  | *Magnolia liliflora*  *Forsythia suspensa*  *Larix gmelini* |

Figure S2. Fluorescence response curves of compounds competitive with GN3. Note: The fluorescence intensity was normalized, and the relative fluorescence intensity of the control group (concentration of probe and drug was 6 µM and 0 µM, respectively) was 100

Table S2 Docking results of EGFR-FKA interactions

| mode | Affinity (kcal/mol) | rmsd 1.b. | rmsd u.b |
| --- | --- | --- | --- |
| 1 | -7 | 0.0000 | 0.0000 |
| 2 | -6.7 | 2.804 | 4.955 |
| 3 | -6.7 | 2.623 | 4.231 |
| 4 | -6.6 | 1.669 | 7.639 |
| 5 | -6.5 | 4.347 | 6.485 |
| 6 | -6.5 | 4.495 | 8.055 |
| 7 | -6.4 | 2.116 | 8.079 |
| 8 | -6.3 | 3.632 | 5.322 |
| 9 | -6.2 | 2.660 | 8.364 |
